# Use of Symptom Checklist 90 for exploring psychological factors among the parents of children hospitalized for burn injuries in Shanghai: a cross-sectional study

**DOI:** 10.1038/s41598-022-25470-1

**Published:** 2022-12-05

**Authors:** Shijie Chen, Qiuping Jiang, Yin Zhang, Changjuan Zeng

**Affiliations:** 1 Department of Nursing, Shanghai Ninth People’s Hospital, Shanghai Jiao Tong University School of Medicine, No. 639, Zhizaoju Road, Huangpu District, Shanghai, 200011 China; 2 Department of Ophthalmology, Shanghai Ninth People’s Hospital, Shanghai Jiao Tong University School of Medicine, No. 639, Zhizaoju Road, Huangpu District, Shanghai, 200011 China; 3 Shanghai Key Laboratory of Orbital Diseases and Ocular Oncology, Shanghai, 200011 China; 4 Shanghai Jiao Tong University School of Nursing, Shanghai, 200025 China; 5 Present Address: Clinical Research Unit, Shanghai Ninth People’s Hospital, Shanghai Jiao Tong University School of Medicine, No. 639, Zhizaoju Road, Huangpu District, Shanghai, 200011 China; 6 Present Address: Department of Burn, Shanghai Corps Hospital of the Chinese People’s Armed Police Force, No. 831, Hongxu Road, Changning District, Shanghai, 201103 China; 7 Present Address: Department of Burn, Ruijin Hospital, Shanghai Jiao Tong University School of Medicine, No. 197, Ruijin 2nd Road, Huangpu District, Shanghai, 200025 China

**Keywords:** Trauma, Psychology, Health care

## Abstract

To better improve the conditions for the recovery of children with burn injuries, timely understanding of the psychological status of parents is important. A cross-sectional survey on it using convenience sampling was conducted at two hospitals. Besides basic information, the Symptom Checklist 90, Eysenck Personality Questionnaire, Social Support Rate Scale, and Simplified Coping Style Questionnaire were used, and the key factors were identified via multivariate linear regression analysis and path analysis. A total of 196 guardians were recruited, 180 valid and completed questionnaires were obtained, including 58 men (32.2%) and 122 women (67.8%), and their average age was 30.3 years (standard deviation = 7.6). Of these, 151 participants (83.9%) were parents. Multivariate analysis revealed that children’s age, parent gender, *P* score, negative coping style, and religion were the main factors that affected parents’ psychology. Moreover, path analysis showed that P score, children’s age, and negative coping style had the greatest impact on the total average score. These results suggest that during hospitalization, the following three factors should be focused on: older children, higher parental psychoticism, and increased negative coping style.

## Introduction

As a vulnerable group, children are prone to accidents owing to their limited cognitive ability to predict or be aware of danger^1,2^. Preventable accidental injuries remain a leading cause of death and acquired disability in children^3^. The growth of children worldwide is threatened by many factors, such as violence, epidemics, and other adverse factors. These factors can have important consequences for the future of families and societies^4^. Burns are one of the major destructive injury forms that endanger the life and health of humans^5^. Burns are also a global public health problem, accounting for an estimated 180,000 deaths each year. The majority of burns occur in low- and middle-income countries, with nearly two-thirds occurring in the African and South-East Asian regions^6^. Much of the research on burn-related injuries has focused on pediatric populations^7,8^. Most burns occur at home with children aged < 3 years while playing; liquids and hot objects were the main causes of the burns^2,9^. Burns not only cause physical and mental harm to children but also bring a heavy economic burden to families. The hospitalization costs of burn victims are high and vary among countries^10^. In the US, the cost of primary care for an inpatient with burns is $3000–$5000 per day, which is double the cost of an average inpatient, and the costs are increasing faster^11^. In the UK, the average cost per hospital admission for burns is approximately $3700^11^. Burns are associated with prolonged hospital stays and mental stress in not only developed countries (such as Europe and the US) but also developing countries. They are a serious health problem, particularly among the economically and socially deprived populations^12^. Burns, with prolonged recovery period and complex morbidity, can disrupt the ability of family members to focus on their work and place a heavy financial burden on the family, adding to high-cost rehabilitation^13^. In addition to financial pressure, burn injuries can cause great psychological pressure on parents. Rizzone et al.^14^ found that 72% of parents reported posttraumatic stress symptoms within the subsequent 6 months of their children’s burn injury incident and that 56% of these parents continued experiencing these symptoms even years after the event^15^. In particular, during the hospitalization of children with burns, their parents have varying degrees of anxiety and depression^16,17^. Moreover, the psychological status of the parents worsens as the degree of the child’s burn increases^18^. As children with burns are generally cared for by their parents, parents’ mentality plays an important role in children’s psychological status and recovery after burns. Thus, medical staff needs to provide necessary psychological intervention to the parents of children with burns in addition to the optimal treatment of children. Therefore, this study aimed to investigate the psychological status of parents and analyze the relationship between these factors to capture key information via a survey, which will simplify future surveys. This will help medical staff identify and quickly intervene with regard to factors that affect the psychological status of parents, adjust the psychological status of parents, and provide more favorable conditions for the psychosomatic recovery of children with burns during hospitalization and even after they return home, thereby shortening the recovery time of these children.

## Methods

### Participants

A cross-sectional survey, which is a type of observational study that analyzes data from a population (or a representative subset) at a specific point in time, was conducted in the present study^19^. From January 2016 to December 2018, the parents of patients who met the admission criteria and admitted to the Department of Burn, Ruijin Hospital Affiliated to Shanghai Jiao Tong University School of Medicine and Department of Burn, Shanghai Corps Hospital of the Chinese People’s Armed Police Force. The inclusion criteria were as follows: parents of inpatient children with burns (age range, 0–18 years); age ≥ 18 years; hospitalization of the child for the first time; hospitalization of the child for ≥ 7 days; clear consciousness with no history of mental illness or severe cognitive impairment; and willingness to participate in this study. The exclusion criteria included the following: unable to take care of themselves and recent history of a major family accident or acute psychological trauma. During the study period, all children and parents who met the inclusion criteria during hospitalization were asked if they were willing to participate in the study, and questionnaires were completed after obtaining their informed consent. Questionnaires missing important information such as gender, age, and other basic information or incomplete questionnaires that affected the calculation of the questionnaire score were considered invalid. The present study was an exploratory analysis of the relationship between factors that influence parental psychology via a questionnaire survey.

A total of 196 parents of children hospitalized with burns were recruited using the convenience sampling method, and 180 valid and completed questionnaires were obtained. The mean age of the parents surveyed was 30.3 years (standard deviation [SD] = 7.6), and they were predominantly female and mostly nonreligious. As the study was an observational study, it was performed in accordance with the STROBE guidelines, which stands for an international, collaborative initiative of epidemiologists, methodologists, statisticians, researchers, and journal editors involved in the conduct and dissemination of observational studies^20^.

### Questionnaires

The basic information of children with burns and their parents, Symptom Checklist 90 (SCL-90), Eysenck Personality Questionnaire (EPQ), Social Support Rate Scale (SSRS), and Simplified Coping Style Questionnaire (SCSQ) were completed and obtained from the participants.

#### Basic situation

The details of the gender, age, nationality, relationship with the child, religion, marital status, occupation, professional qualifications, and monthly income of the parents were obtained using a questionnaire completed by the parents.

The details of the gender, age, school level, number of pediatric hospital visits because of trauma in the past year, and number of children in family of children with burns were obtained using another questionnaire filled by the parents. Other details of time between the incidence of burns and hospital visit and the degree of burns were completed by a nurse.

#### SCL-90

SCL-90, compiled by American psychologist Derogatis et al. in 1973^21^, is used to test whether parents may have a mental disorder and its severity. It was translated into Chinese by Wang in the 1980s^22^. The scale includes 90 items with a total of 10 subscales, namely somatization, obsessive–compulsive symptoms, interpersonal sensitivity, depression, anxiety, hostility, phobic anxiety, paranoid ideation, psychoticism, and other additional items. The total average score of SCL-90 is also the total symptom index. Increase in the total average score indicates more obvious symptoms. The calculation method is to divide the total score by 90. The present study used the Chinese version of SCL-90, which has been cited in China for > 30 years and has good reliability and validity. Cronbach’s α coefficient of the total scale is 0.97, Cronbach’s α coefficient of each subscale is > 0.69, and the test–retest reliability is > 0.7^23,24^.

#### EPQ

To understand parental personality factors, EPQ, which was developed by Chen et al.^25–27^, was used. China revised this scale in the 1980s to make the Chinese version of EPQ more suitable for Chinese people; this version was adopted in this study. The scale has 88 items and contains three dimensions, including the four subscales of extraversion (E), neuroticism (N), psychoticism (P), and lie (L), with good reliability and validity^25,26,28^.

#### SSRS

To understand the characteristics of parents’ social support and its relationship with mental health level, mental illness, and various physical diseases, SSRS, developed by Xiao et al.^29,30^, was used. The scale has a total of 10 items, including three dimensions of objective support (three items), subjective support (four items), and use of social support (three items). The authors of this scale, Xiao et al., evaluated SSRS, and the results revealed that the total score reliability is 0.92 and the reliability of each item is between 0.89 and 0.94, indicating that the questionnaire has good test–retest reliability^30^.

#### SCSQ

To understand the relationship between parental coping styles and psychosomatic health, SCSQ was used^31^. The scale comprises two subscales with 20 items: positive coping style (12 items) and negative coping style (8 items). Items were measured on a 4-point Likert scale (0 = never, 1 = occasionally, 2 = sometimes, and 3 = frequently)^32^. This scale has good reliability and validity: Cronbach’s α coefficient of the scale is 0.90, Cronbach’s α coefficient of the positive coping scale is 0.89, Cronbach’s α coefficient of the negative coping scale is 0.78, and the test–retest correlation coefficient is 0.89^31^.

### Data analysis

Data management and statistical analysis were performed using SPSS 26.0 and AMOS 24.0 software. Considering that very few variables had missing values and the proportion of missing data was small, the data were analyzed regardless of them. Percentages described the qualitative data and ${\overline{\text{x}}}\, \pm \,{\text{s}}$ described the quantitative data that conformed to normal distribution. Owing to the large number of influencing factors collected, univariate analysis was performed to analyze the influence of relevant factors on parents’ psychology. The factors with *P* < 0.05 were identified from the univariate analysis results and entered into multivariate analysis. Multivariate analysis was performed using multiple linear regression and stepwise method to obtain the relationship between related factors. AMOS 24.0 was used to analyze the path and plot the path analysis diagram. All tests were two-sided, and *P* < 0.05 indicated statistical significance.

### Ethics committee approval

Ethics committee approval for the study was received from Ruijin Hospital Affiliated to the Shanghai Jiao Tong University School of Medicine Ethics Committee (IRB ID: (2015) Clinical Ethics Review No. 101), and the research materials were used only for academic research.

### Informed consent

Consent was obtained before inclusion in the study. Participation in the study was voluntary, and participants could withdraw from the study at any time during the study.

## Results

A total of 180 valid questionnaires were obtained, of which 29 were from the Department of Burn of Ruijin Hospital and 151 were from the Department of Burn, Shanghai Corps Hospital of the Chinese People’s Armed Police Force. Each participant was required to complete five questionnaires: one basic information survey and four psychological questionnaires. The average age of the surveyed guardians was 30.3 years (SD = 7.6). There were 58 men (32.2%) and 122 women (67.8%). Among the participants, the most common relationship with the children was parents (151 [83.9%]). The average age of the children surveyed was 4.0 years (SD = 3.8): 100 males (55.6%) and 80 females (44.4%). The degree of children’s burns was mainly mild and moderate, accounting for 41.1% and 37.8%, respectively (see Table 1 for details).

**Table 1:** Characteristics of the study population and univariate analysis results (n = 180).

Characteristics	Value	*P* value^a^
**Parent gender, n (%)**		
Male	58(32.2)	**0.003**
Female	122(67.8)	
**Nationality, n (%)**		
Han	177(98.3)	**0.006**
Others	3(1.7)	
**Relationship, n (%)**		
Father/mother	151(83.9)	0.248
Grandfather/grandmother	18(10.0)	
Maternal grandfather/grandmother	3(1.7)	
Others	8(4.4)	
**Religion, n (%)**		
None	140(77.8)	**0.001**
Buddhism	17(9.4)	
Islam	5(2.8)	
Christianity	7(3.9)	
Taoism	1(0.6)	
Others	10(5.6)	
**Occupation, n (%)**		
Employed	156(86.7)	**0.012**
Retirement	1(0.6)	
Student	12(6.7)	
Unemployed	11(6.1)	
**Professional qualifications, n (%)**		
None	90(50.0)	**0.002**
Elementary	28(15.6)	
Intermediate	44(24.4)	
Advanced	17(9.4)	
Missing	1(0.6)	
**Marital status, n (%)**		
Other (single, divorce, widowed, or any other)	37(20.6)	**0.002**
Married	143(79.4)	
**Monthly income (RMB), n (%)**		
< 3000	39(21.7)	0.415
3000–5000	74(41.1)	
5001–10,000	47(26.1)	
> 10,000	20(11.1)	
**Children gender, n (%)**		
Male	100(55.6)	0.472
Female	80(44.4)	
**School level, n (%)**		
None	96(53.3)	** < 0.001**
Kindergarten	46(25.6)	
Primary school	25(13.9)	
Middle school	7(3.9)	
High school	3(1.7)	
Missing	3(1.7)	
**Burn degree, n (%)**		
Mild	74(41.1)	0.07
Moderate	68(37.8)	
Severe	18(10)	
Extra severe	2(1.1)	
Missing	18(10.0)	
**Parents age, years: mean ± SD**	30.3 ± 7.6	0.653
**Children age, years: mean ± SD**	4.0 ± 3.8	< **0.001**
**Time between burn incidence and hospital visit, h: mean ± SD**	2.6 ± 3.7	0.127
**Number of pediatric hospital visits for trauma last year, time: mean ± SD**	3.1 ± 1.2	0.200
**Number of children in the family, number: mean ± SD**	1.3 ± 0.6	**0.022**

Significant values are in [bold].

The abbreviation for China Yuan is RMB.

^a^The result of univariate analysis in which the dependent variable was total average score (SCL-90).

The results of the four psychological questionnaires were as follows. In EPQ, the average score of the P scale was 8.62 points (SD = 3.99), average score of the E scale was 11.98 points (SD = 4.03), average score of the N scale was 12.76 points (SD = 6.12), average score of the L scale was 9.92 points (SD = 4.99), and Cronbach’s α coefficient was 0.92. In SSRS, the average total score was 38.75 points (SD = 10.39), the average score for objective support was 7.84 points (SD = 3.85), the average score for subjective support was 23.54 points (SD = 6.42), and the average score for use of social support was 7.38 points (SD = 2.62). Its Cronbach’s α coefficient was 0.88. In SCSQ, the average score of the positive coping style was 28.11 points (SD = 9.65), the average score of the negative coping style was 15.62 points (SD = 5.56), and its Cronbach’s α coefficient was 0.94. Finally, in SCL-90, the total average score was 1.67 points (SD = 0.75) and its Cronbach’s α coefficient was 0.98. The reliability test results of these four scales suggested that the internal consistency of the four scales is very high (see Table 2 for details).

**Table 2:** Result of scales and univariate analysis.

Scale	Value (mean ± SD)	Cronbach’s α	*P* value^a^
**Eysenck Personality Questionnaire**			
P score	8.62 ± 3.99	0.92	** < 0.001**
E score	11.98 ± 4.03		0.431
N score	12.76 ± 6.12		** < 0.001**
L score	9.92 ± 4.99		**0.008**
**Social Support Rate Scale**			
Total score	38.75 ± 10.39	0.88	**0.029**
Objective support	7.84 ± 3.85		0.073
Subjective support	23.54 ± 6.42		0.117
Use of social support	7.38 ± 2.62		**0.031**
**Simplified Coping Style Questionnaire**			
Positive coping style	28.11 ± 9.65	0.94	0.942
Negative coping style	15.62 ± 5.56		< **0.001**
**Symptom Checklist 90**			
Total average score*	1.67 ± 0.75	0.98	/

Significant values are in [bold].

^a^The result of univariate analysis.

*The dependent variable.

In this study, the total average score of SCL-90 was used as the dependent variable and the remaining survey factors were used as independent variables. Both univariate and multivariate analyses were performed using multiple linear regression, and the results of univariate analyses are detailed in Tables 1 and 2. The following factors were significant in univariate analysis: parent gender, nationality, marital status, occupation, professional qualifications, religion, children age, school level, number of children in the family, negative coping style (SCSQ), total score (SSRS), use of social support (SSRS), P score (EPQ), N score (EPQ), and L score (EPQ). These factors were included in multivariate analysis in which dummy variables were set for disordered multicategory variables and the stepwise method (entry, 0.05; removal, 0.10) was used to screen variables. The following variables were entered in the regression equation: children’s age, parent gender, P score (EPQ), negative coping style (SCSQ), and religion. The multivariate analysis results are shown in Table 3. For the obtained regression equation, R^2^ = 0.413 and adjusted R^2^ = 0.355. In the regression equation, the dummy variable of religion for nonreligious people, except the adherent of Christianity, had an effect on the total average score, and the other dummy variables were not significant.

**Table 3:** Multiple linear regression model for contributing factors of total average score (SCL-90).

Model summary	R square = 0.413		Adjusted R square = 0.355		
Contributing factors	B	SE	Beta	t	*P*
Children age	0.043	0.013	0.227	3.309	**0.001**
Parent gender	0.238	0.107	0.150	2.227	**0.028**
P score (EPQ)	0.043	0.013	0.234	3.286	**0.001**
Negative coping style (SCSQ)	0.032	0.009	0.244	3.591	< **0.001**
Buddhism (religion)*	0.287	0.228	0.098	1.258	0.210
Islam (religion)*	0.488	0.303	0.115	1.612	0.109
Christianity (religion)*	0.591	0.259	0.164	2.281	**0.024**
Taoism (religion)*	1.270	0.660	0.136	1.925	0.056
Others (religion)*	0.087	0.205	0.029	0.427	0.670

Significant values are in [bold].

*The dummy variable of religion; the reference was none (religion).

**Figure 1:**
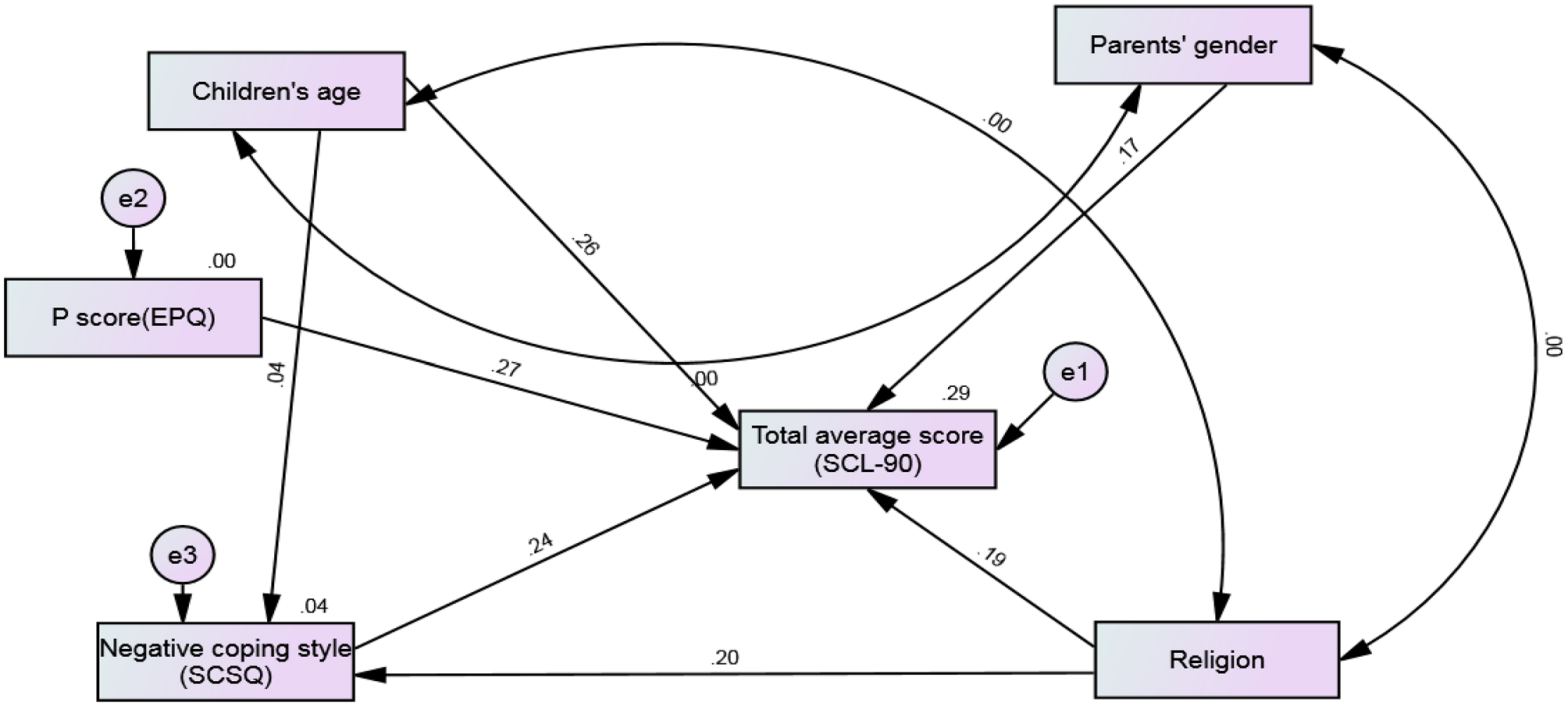
Path analysis diagram and final model of total average score (SCL-90).

The significant factors obtained using multivariate analyses were determined and established after correction and adjustment, and the path analysis model diagram was established (see Fig. 1 for details). For the obtained path analysis model, chi-square = 16.51; degrees of freedom = 8; *P* = 0.036; and the fit index of the model was GFI = 0.969, AGFI = 0.919, and RMSEA = 0.08, suggesting that the model fit satisfactorily. The specific path coefficients (after normalization) are shown in Table 4. The three factors that had the greatest impact on the total average score were P score (EPQ), children’s age, and negative coping style (SCSQ) in the descending order.

**Table 4:** Standardized estimates of associations between variables.

Dependent	Variable	Standardized estimate	C.R	*P*
Negative coping style (SCSQ)	Religion	0.197	2.594	**0.009**
Negative coping style (SCSQ)	Children age	0.043	0.563	0.573
Total average score (SCL-90)	Parent gender	0.175	2.67	**0.008**
Total average score (SCL-90)	Negative coping style (SCSQ)	0.24	3.595	***
Total average score (SCL-90)	Religion	0.192	2.882	**0.004**
Total average score (SCL-90)	Children age	0.263	4.02	***
Total average score (SCL-90)	*P* score (EPQ)	0.267	4.074	***

Significant values are in [bold].

***P < 0.001.

## Discussion

In the present study, using multivariate linear regression and path analyses, the following three factors were found to affect the psychology of the parents of children with burn injuries: high P score (higher psychoticism), older children, and more negative coping style. Parents’ psychological status and severity were assessed using SCL-90 because it is one of the most comprehensive and widely used scales for measuring a range of psychological and psychiatric symptoms internationally^33^. The present survey study showed that the parents of children with burn injuries had obvious psychosomatic symptoms, and 31% of them had a total average score of > 2 points in SCL-90.

The multivariate analysis results of linear regression revealed that the factors affecting the total average score were children’s age, parent gender, P score (EPQ), negative coping style (SCSQ), and religion. The obtained equation with R^2^ = 0.456 and adjusted R^2^ = 0.381 indicated that the model fit was satisfactory (Table 3).

Several studies have shown that the risk of burns decreases with the increasing age of children^34,35^. In line with these studies, in the present study, 81.4% of the included participants were preschool children under the age of 7 years. In multivariate analysis, with the increase in children’s age, the total average score also increased, i.e., the psychological symptoms of parents increased. This may be attributed to the possibility of burns gradually decreasing with increasing age. Thus, it is more likely to have an impact on parents’ psychology when their children get burn injuries. In line with the current study results, other studies have indeed confirmed that the age of the child had the same effect on parents’ psychology^36^.

The mean age of the women in this study was 29.04 years (SD = 7.41), and they were relatively young parents. In the multivariate analysis, with respect to the gender of parents, female parents had higher total average scores and more obvious psychological symptoms, which is consistent with related research as follows. During coronavirus disease 2019, Maggi et al., in a study conducted in Italy, concluded that younger individuals and females experienced more severe symptoms of depression and anxiety^37^. A Pakistani survey study conducted by Rabbani et al. revealed that compared with men, women were more anxious during the pandemic and they experienced a disproportionate burden of the psychological and social impact of the pandemic^38^. Through their study in China, Xie et al. concluded that women and younger professionals appeared to be affected most by job demands and psychological distress^39^. Additional relevant studies have shown that young women suffering from the stressful times of bullying tend to have a higher risk of suicidal attempts and nonsuicidal self-injury^40,41^. Moreover, as per a study, female parents were prone to psychological problems and that among the parents of children of all ages, mothers were more likely to suffer from mental illnesses than fathers^42^. Another study showed that there were significantly more women than men in psychiatric specialist visits, which may be attributed to women being more introverted, calm, solitary, indifferent with others, pessimistic, tense and anxious, more thoughtful, excitable, and difficult to calm down and they often complained of various physical discomfort and other symptoms^43^.

The P subscale in EPQ is one of the dimensions of people’s personality. People with high *P* scores are inclined toward being cruel, inhumane, socially indifferent, hostile, aggressive, inconsiderate of danger, and intolerant^44^. Linear regression showed that the higher the *P* scores, the higher were the total average scores, indicating that parents with higher *P* scores had a higher propensity toward being troublesome, belittling, acting disruptively, and lacking in empathy^44^, thereby having a negative impact on the recovery of a child with burns. This is consistent with the findings of previous studies, which revealed that mentality in the personality dimension had a greater impact on mental health^43,45^.

Joff et al. believed that coping is an individual’s conscious, purposeful, and flexible adjustment behavior to changes in the real environment^46^. According to the results of existing theories and practical observations, people’s coping styles can be relatively divided into “positive” and “negative” coping styles when they experience setbacks. SCSQ, a simple and easy to understand questionnaire, was developed by classifying various coping styles according to their common characteristics^31^. The negative coping style items in SCSQ mainly reflect the characteristics of negative coping. When the negative coping score is high, the psychological problem or symptom score is also high. In linear regression analysis, a higher negative coping score increased the impact on the total average score. This represented a greater impact on parents’ psychology.

Religion is a very complex factor, and while religion can represent a powerful source of comfort, hope, and meaning, it is often intricately entangled with neurotic and psychotic disorders, sometimes making it difficult to determine whether it is a resource or liability^47^. Therefore, it is difficult to draw a conclusion on whether the psychological impact of religion is beneficial or harmful, and it may vary as per the situation. Multivariate analysis showed that compared with people without religion, people who followed Christianity had a higher total average score of SCL-90. However, other religions were not statistically significant, reflecting the complexity of the religion factor itself. Bentzen et al. suggested that in times of crisis, humans tend to turn to religion for comfort and explanation, and their research, which included 107 countries, demonstrated that the coronavirus disease 2019 crisis resulted in a massive rise in the intensity of prayer, people pray to cope with adversity^48^. Wang et al. argued that in China, individuals who are affiliated with a religion are considered a marginalized population. Marginalization may lead to psychological strains, further increasing the risk of suicidality^49^. Therefore, it has been hypothesized that after a burn injury in children, their parents may seek religious support and some nonreligious people may turn to religion^17^. In addition, some may already manifest a psychological disorder or psychological problems before seeking religious support^17^. These results were also observed in the present study, which showed high SCL-90 scores among religious people or high SCL-90 scores owing to the psychological pressure felt by marginalization due to religious beliefs. Moon et al., however, suggested that a critical eye be applied to these questions of specialness and concluded that although it is clear that religion is psychologically important, there is no strong evidence yet that it is psychologically special, with the possible exception of its effects on health^50^. Thus, the complexity of religion as a factor was observed in the present study, and subsequent studies should validate the effect of religion.

Regression analysis focuses on the relationship between dependent and independent variables; however, it pays less attention to the correlation mechanisms that may exist among all the variables^51^. By contrast, path analysis emphasizes identifying correlation in the model and total determination in terms of model parameters and then plotting the path diagram^52,53^. Therefore, based on multivariate regression analysis, path analysis can be used to understand the relationship between related factors more comprehensively and provide a complete picture between the various factors. Moreover, path diagram, a graphical representation of a system of simultaneous equations, expresses the assumed relationship more clearly than equations^54^. The final path model obtained in the present study fit well, and the five influencing factors identified using linear regression also had significant effects on the total average score in the path analysis (Table 4 and Fig. 1). The relationship between the variables was obtained after the path coefficients were standardized among the variables. The variable with the greatest contribution to the total average score and > 0.2 was the P subscale whose path coefficient was 0.267, followed by the age of children with 0.263 and negative coping with 0.24. Therefore, these three aspects can be considered the psychological influencing factors of parents of children with burns, and parents with these three factors should be evaluated. Moreover, the age of children had an impact on negative coping, but its path coefficient was 0.04, and negative coping had already been the focus of our attention, so the impact of children’s age on negative coping was not the main concern.

In clinical practice, medical staff should be instructed to pay attention to the parents of older children with burn injuries and evaluate their psychological status. The use of the P scale in EPQ and the negative scale in SCSQ, which were simplified and analyzed in this study, should be encouraged. Based on these factors, key objects can be identified and psychological counseling can be offered in time to provide favorable conditions for their children’s psychological and physical recovery after burn injuries.

## Limitation

The current study adopted the method of combining multivariate regression analysis and path analysis, which not only identified the influencing factors but also clarified the relationship between them. Combining both analyses recognized the key information in the survey and simplified the evaluation. However, the following limitations should be considered. First, in this study, 180 valid surveys were collected. Thus, the sample size was not large, but each subject was evaluated as completely as possible. In addition to basic information, four psychological questionnaires were used to comprehensively understand the psychological status of parents and collect relevant factors that may affect it. Second, two centers were included in the study, with 29 valid surveys from the Department of Burns and Plastic Surgery of Ruijin Hospital and 151 from the Department of Burn, Shanghai Corps Hospital of the Chinese People’s Armed Police Force. However, convenience sampling, which may lack overall representativeness, was used in this study.

## Conclusions

Multivariate linear regression revealed that the high-risk factors affecting parents’ psychology were children’s age, parent gender, P score (EPQ), negative coping style (SCSQ), and religion. Moreover, path analysis showed that older children, parental higher psychoticism, and more negative coping style are main concerns in the parents of children with burns. Thus, medical staff should be guided to intervene in parents’ psychological problems earlier to provide favorable conditions for the better recovery of children with burns.

## Data Availability

The datasets used and analyzed in this study are available from the corresponding author Changjuan Zeng on reasonable request.
